# Protein Kinase C inhibition ameliorates functional endothelial insulin resistance and Vascular Smooth Muscle Cell hypersensitivity to insulin in diabetic hypertensive rats

**DOI:** 10.1186/1475-2840-10-48

**Published:** 2011-06-02

**Authors:** Xiao Lu, James S Bean, Ghassan S Kassab, Mark D Rekhter

**Affiliations:** 1 Department of Biomedical Engineering, Cellular and Integrative Physiology, Surgery, and Indiana Center for Vascular Biology and Medicine, Indiana University Purdue University, Indianapolis, IN 46202, USA; 2 Lilly Research Laboratories, Indianapolis, IN 46285, USA

## Abstract

**Objective:**

Insulin resistance, diabetes, and hypertension are considered elements of metabolic syndrome which is associated with vascular dysfunction. We investigated whether inhibition of protein kinase C (PKC) would affect vascular function in diabetic hypertensive (DH) rats.

**Methods:**

A combination of type 2 diabetes and arterial hypertension was produced in male Sprague Dawley rats by intrauterine protein deprivation (IUPD) followed by high salt diet. At the age of 32 weeks, DH rats were treated for 2 weeks with the angiotensin-converting enzyme inhibitor captopril (Capto, 30 mg/kg), PKC inhibitor ruboxistaurin (RBX, 50 mg/kg) or vehicle (n = 8 per group) and blood pressure was monitored using telemetry. At the end of experiments, femoral arteries were dissected, and vascular reactivity was evaluated with isovolumic myography.

**Results:**

The IUPD followed by high salt diet resulted in significant elevation of plasma glucose, plasma insulin, and blood pressure. Endothelium-dependent vascular relaxation in response to acetylcholine was blunted while vascular contraction in response to phenylephrine was enhanced in the DH rats. Neither Capto nor RBX restored endothelium-dependent vascular relaxation while both suppressed vascular contraction. Ex-vivo incubation of femoral arteries from control rats with insulin induced dose-response vasorelaxation while insulin failed to induce vasorelaxation in the DH rat arteries. In the control arteries treated with endothelial nitric oxide synthase inhibitor L-NAME, insulin induced vasoconstriction that was exacerbated in DH rats. Capto and RBX partially inhibited insulin-stimulated vascular contraction.

**Conclusion:**

These findings suggest that PKC inhibition ameliorates functional endothelial insulin resistance and smooth muscle cell hypersensitivity to insulin, but does not restore acetylcholine-activated endothelium-dependent vasodilation in DH rats.

## Introduction

Insulin resistance, type 2 diabetes, and hypertension are often clustered as part of metabolic syndrome [1]. Endothelial dysfunction is a salient and, likely a unifying feature of metabolic syndrome that translates systemic risk factors into vascular pathology [2]. It has been recently recognized, however, that endothelial dysfunction is far more complex than blunted endothelium-dependent vasodilation in response to acetylcholine or blood flow (shear stress). Specifically, endothelial insulin resistance appears to be one of the key mechanisms of vascular dysfunction in metabolic syndrome and a major cause of atherosclerosis [3-5].

Protein kinase C (PKC) is intimately involved in development of vascular insulin resistance and vascular dysfunction [6]. Ruboxistaurin (RBX), a PKC inhibitor, has been widely used to study vascular function in animal models related to metabolic syndrome and in clinical research [4,7-9]. Many questions regarding vascular effects of PKC inhibition, however, remain unanswered. First, it is unclear whether PKC inhibition uniformly or differentially regulates vascular response to insulin and "classical" vasoreactive substances (e.g., acetylcholine and epinephrine). Second, while the role of PKC in endothelial dysfunction is well documented, less is known about smooth muscle cell (SMC) response to insulin. Third, available data related to vascular insulin resistance is largely of biochemical nature and there is a lack of complementary data on vascular function. Fourth, vascular insulin resistance has been primarily studied in models of metabolic syndrome driven by genetic defects that may potentially skew the results and jeopardize clinical translation.

Here, we analyzed the influence of pharmacological PKC inhibition on vascular function in a model that combines diabetes and hypertension, while angiotensin-converting enzyme (ACE) inhibitor Captopril (Capto) was used as a positive control in the blood pressure portion of the study [10]. We utilized the rat model of intrauterine protein deprivation (IUPD) followed up by the long term administration of high salt diet as described previously [2,10,11]. In this model, diabetes and hypertension are induced by a combination of relevant non-genomic factors that are applied in the pre- and post-natal periods which reflect the complex environmental origins of metabolic syndrome.

## Materials and methods

The animal experiments were conducted in accordance with the guidelines of Institute of Laboratory Animal Research Guide, Public Health Service Policy, Animal Welfare Act, and an approved IACUC protocol of Eli Lilly and Company.

### In vivo manipulations

**Figure 1:**
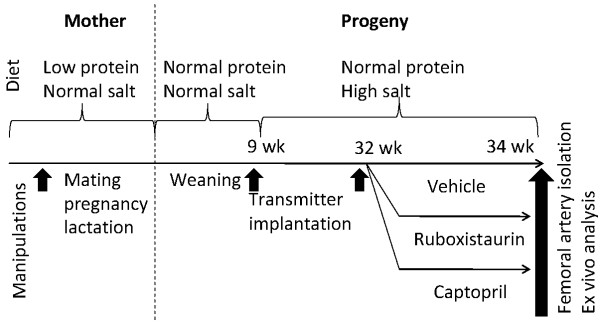
**Schematic of the study design**.

A total of 24 male rats that underwent intrauterine protein deprivation (IUPD) and age matched control rats were obtained from Charles River Laboratories. In brief, IUPD rats were obtained from female Sprague Dawley (SD) rats placed on TD.90016 diet (6%protein) 2 weeks before mating, during pregnancy, lactation, and until weaning (Figure 1). Eight additional control rats were derived from female SD rats maintained on normal rodent chow (20% protein).

All IUPD rats underwent pressure transmitter implantation surgery after a 2 week acclimation period (see schematic of study design in Figure 1). The transmitters (model TA11PA-C40; Data Sciences International, St. Paul, MN) were surgically implanted in iliac artery using a method previously described [12]. At age of 9 weeks (Figure 1), all instrumented rats were placed on a high salt (6%) diet (Test Diet 0009386) and became diabetic hypertensive (DH). Two weeks post surgery, baseline blood pressure data were measured. Digitized pressure signals were acquired for 30 s every 10 min using DSI Dataquest IV 2.0 software. Mean pressure was calculated as the arithmetic mean of the sample waveform sampled at a frequency of 500 Hz. The digitized values were stored and manipulated on a Compaq 486/33 MHz computer.

At the age of 32 weeks, DH rats were assigned to one of three groups (n = 8): vehicle, the ACE inhibitor Capto 30 mg/kg or PKC inhibitor RBX 50 mg/kg. Both Capto and RBX were administered as an admixture in the diet used for the controls (Test Diet 0009386) for 2 weeks (Figure 1). The body weights and food consumption were measured weekly.

Plasma samples were collected by cardiac draw at the termination of the study. Glucose, free fatty acid (FFA) and triglyceride levels were analyzed by a Hitachi analyzer model 912 (Roche, Indianapolis, IN). A rat sensitive insulin kit (Millipore Bioscience Division St. Charles, MO) was used to determine insulin levels.

### Isovolumic myography for endothelial and vascular smooth muscle functions

The vascular function data presented below were intended to unravel the potential effects of PKC inhibition on arterial endothelial and SMC function. At termination day, the animals were anesthetized with isofluorane. The common femoral arteries were harvested and the animal was euthanatized with over-dose of anesthesia. The excised vessels were immediately stored in 4°C HEPES physiologic saline solution (HEPES-PSS, pH7.4, in mmol/l: 142 NaCl, 4.7 KCl, 2.7 HEPES Na, 3 HEPES, 1.17 MgSO_4_, 2.79 CaCl, 5.5 Glucose; HEPES and HEPES Na were purchased from Sigma, while others were purchased from Fisher Scientific, USA). The blood was sampled and the plasma was separated and stored at -80°C. The arterial segment was carefully cleaned from adjacent tissue with the aid of a stereo-dissection microscope. The branches on the artery were ligated and the vessel was allowed to warm up to room temperature (22°C) slowly in 10-15 min. The vessel was transferred to the chamber of isovolumic system and cannulated with connectors and secured with 8-0 suture twice to avoid any leakage. The vessel was warmed up to 37°C slowly (20-25 min) and equilibrated for 40 min at a transmural pressure of 25 mmHg before agonist and antagonist stimulation.

**Figure 2:**
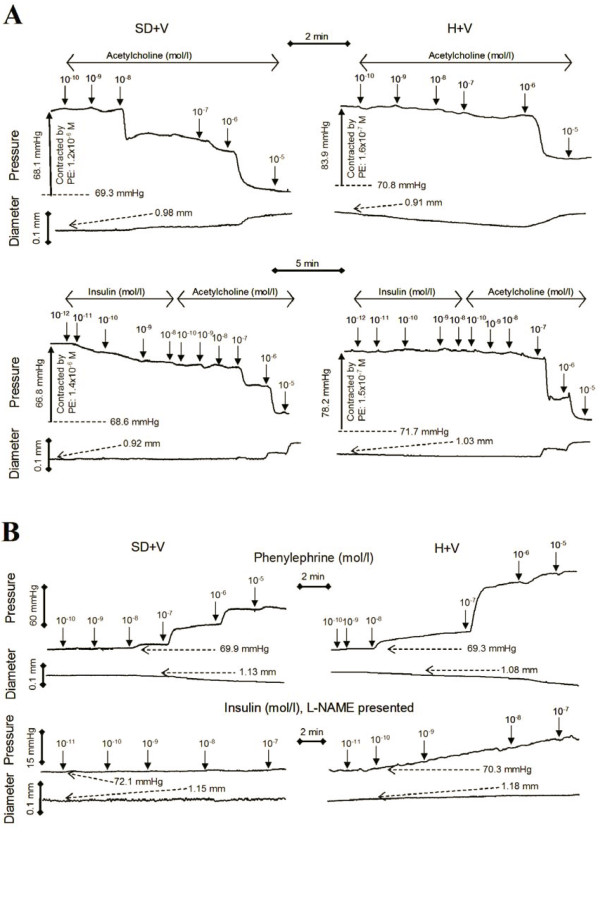
**The typical tracing curves of pressure and diameter versus time during vasoreactivity which were measured by isovolumic myograph**. A: The endothelium-dependent dose-response vasorelaxation. The vessels were contracted by phenylephrine (PE) to approximate pressures and relaxed by a series of doses of acetylcholine (ACh) (top panel) or a series of doses of ACh following by a series doses of insulin (bottom panel). B: The dose-response vasoconstriction in response to PE (top panel) and insulin with presentation of L-NAME (bottom panel).

A novel isovolumic method was recently introduced that has the physiology of pressure myograph with sensitivity of wire myograph to evaluate the vasoreactivity of small and large vessels [13,14]. Briefly, the femoral vessel was cannulated on both ends in a bath with HEPES-PSS and incubated at 37°C. The vessel was stretched to *in situ *length and pre-loaded at physiologic pressure of 70 mmHg and pharmacologically induced contraction or relaxation with both ends closed. The contraction or relaxation of the vessel wall against the luminal fluid increases or decreases the pressure, respectively. The pressure was measured with a pressure transducer (Mikro-Tip SPR-524, Millar Instruments, USA) and external diameter was measured with a dimensional tracer (DiamTrak 3+, Australia). The typical tracing curves of pressure and diameter during vasoreactivity were represented in Figure 2.

### Endothelium-dependent relaxation to Acetylcholine

The arterial segment was contracted to an approximate transluminal pressure (150 ± 15 mmHg) with a submaximal dose of phenylephrine (10^-7 ^to 10^-6 ^mol/L). Thereafter, the endothelium-dependent dose-response relaxation was induced with a series of doses of acetylcholine (ACh) from 10^-9 ^to 10^-6 ^mol/L.

### Insulin- and acetylcholine-induced vasorelaxation

The arterial segment was contracted to transluminal pressure of 150 ± 15 mmHg with a submaximal dose of phenylephrine (10^-7 ^to 10^-6 ^mol/L). Insulin was added to the bath at concentrations of 10^-11 ^to 10^-8 ^mol/L. After insulin induced vasorelaxation, acetylcholine dose-response (10^-9 ^to 10^-6 ^mol/L) was administered following maximal concentration of insulin.

### Relaxation in response to exogenous nitric oxide (NO)

The arterial segment was contracted to an approximate transluminal pressure (150 ± 15 mmHg) with a submaximal dose of phenylephrine (10^-7 ^to 10^-5 ^mol/L). The endothelium-independent relaxation to verify the sensitivity of vascular smooth muscle to NO was induced with a series of doses of sodium nitroprusside (SNP): 10^-9 ^to 10^-5 ^mol/L.

### Vasoconstriction to adrenergic agonist

The dose response contractile tension to phenylephrine was performed at a series of dose: 10^-8 ^to 10^-5 ^mol/L.

### Insulin-induced vasoconstriction in the absence of NO

The vessel segment was incubated with the endothelial NO synthase (eNOS) inhibitor *N*^*ω*^-nitro-L-arginine methyl ester (L-NAME, 10^-5 ^mol/l) for 30 min. Insulin was added to the bath at concentrations of 10^-11 ^to 10^-8 ^mol/L.

### Baseline Vasoreactivity

Non-receptor specific contraction to potassium chloride (KCl, 60 mmol/L) and vasorelaxation in response to calcium-free were measured to verify the calcium-mediated contraction and relaxation of vascular smooth muscle.

### Data analysis and Statistics

The circumferential tension of the vessel at every dose was calculated by Laplace's equation, tension = pressure × inner radius. The inner radius was calculated using the incompressibility assumption [14]. The % decrease in tension was calculated by the equation: *%Relaxation *= (*T*_*d *_*- T*_*i*_)**/**(*T*_*max *_*- T*_*i*_)× 100, where, *T*_*d*_, *T*_*i*_, and *T*_*max *_are the tension at every dose of vasodilators (*T*_*d*_), physiological level (*T*_*i*_), and maximum tension (*T*_*max*_) at at a sub-maximal concentration of phenylephrine, respectively. The agonist concentration that produced 50% of the maximal vasorelaxation (IC_50_) and vasoconstriction (EC_50_) was calculated. Data were presented as mean ± standard deviation and significant differences between groups were determined by Student's *t-*test (two-tailed distribution, two-sample unequal variance). Significant differences between the dose-dependent groups were determined by Two-way ANOVA between groups (following Bonferroni test). A probability of p < 0.05 was considered to be indicative of a statistically significant difference.

## Results

### Systemic changes

**Figure 3:**
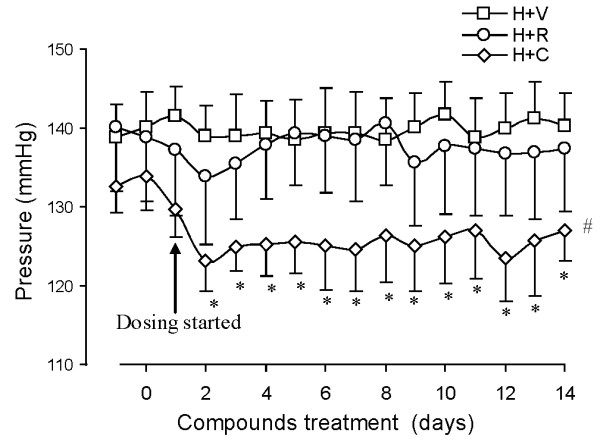
**High salt fed IUPD rats develop arterial hypertension (P < 0.05 in comparison with SD rats)**. The mean pressure of SD rats was 97 ± 11 mmHg. Capto treatment significantly decreased blood pressure (P < 0.05), while RBX did not affect blood pressure (P > 0.05). n = 7; *: P < 0.05 vs. vehicle treated DH rats at the time point. #: P < 0.05 vs. RBX treated DH rats using two way ANOVA.

Body weight and triglycerides were not significantly different in any of the groups (Table 1). Plasma glucose, insulin, and FFA were significantly elevated in DH rats (Table 1). The treatments with Capto and RBX did not have a significant effect on these plasma parameters. Blood pressure (Figure 3) was significantly increased in DH rats in comparison with that in SD rats (97 ± 11 mmHg). As expected, Capto exerted anti-hypertensive effects while RBX treatment did not change blood pressure [10].

**Table 1:** Systemic effects of drug treatment in DH rats

	SD rat	DH rat		
		
	Vehicle	Vehicle	Capto	RBX
Body Weight (grams)	539.2 ± 43.3	573.8 ± 49.1	566.4 ± 46.6	566.1 ± 45.6
Glucose (mg/dl)	129.4 ± 8.65	284.5 ± 38.5*	278.7 ± 28.7*	284.0 ± 22.7*
Trigl yceride (mg/dl)	122.5 ± 18.14	135.2 ± 48.5	130.8 ± 50.8	98.2 ± 42.2
Insulin (mg/dl)	1.42 ± 0.462	2.23 ± 1.97*	2.07 ± 1.46*	1.50 ± 0.787
FFA (mg/dl)	223.6 ± 84.2	660.7 ± 170.6*	483.5 ± 152.6*	462.7 ± 145.4*

Notes: FFA: free fatty acid; Capto: captopril treatment; RBX: ruboxistaurin treatment; SD: Sprague Dawley rat; DH: diabetic hypertension. * indicates statistical difference in comparison with control (P < 0.05).

### Endothelial function

**Figure 4:**
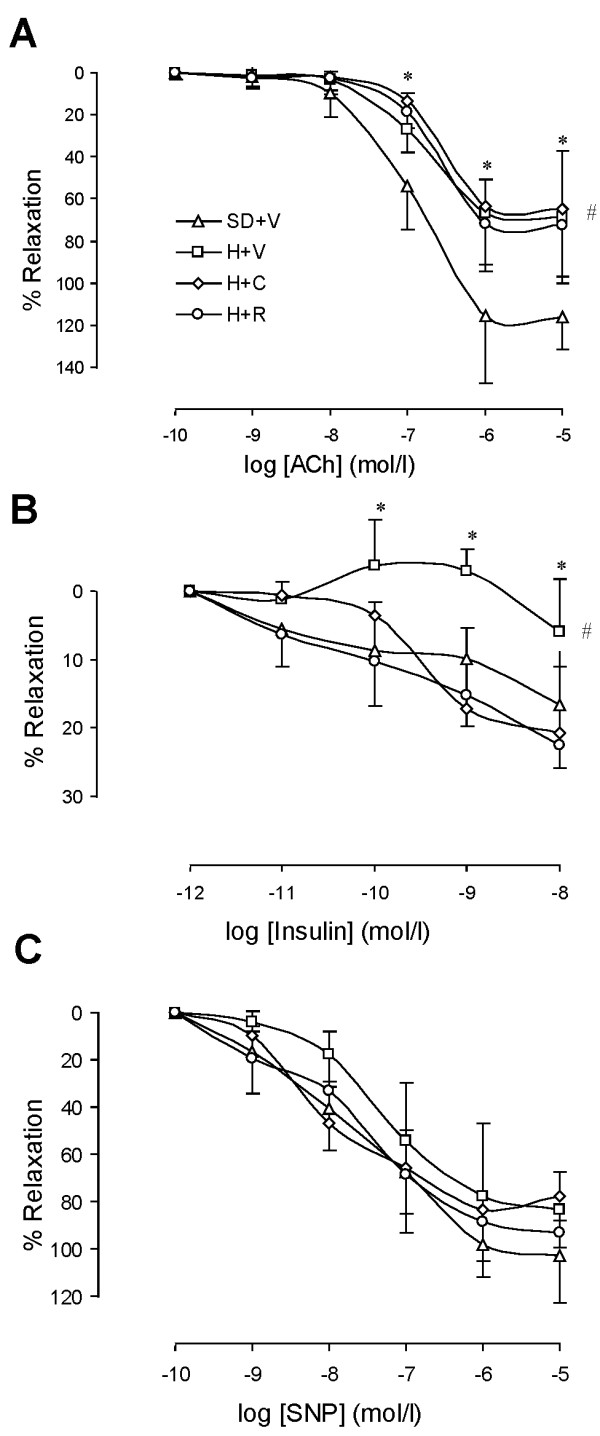
**Analysis of vasorelaxation**. A: Endothelium-dependent vasorelaxation in response to acetylcholine while vessel was pre-constricted with phenylephrine (PE). SD+V: Vehicle treated Sprague-Dawley rats. H+V: Vehicle treated DH rats. H+C: Captopril treated DH rats. H+R: RBX treated DH rats. B: Vascular relaxation in response to insulin while vessel was pre-constricted with PE. C: Endothelium-independent vasorelaxation in response to SNP while vessel was pre-constricted with PE. n = 7; *: P < 0.05 vs. vehicle treated of SD rats at the doses. #: P < 0.05 vs. vehicle treated SD rats using ANOVA.

The endothelium-dependent vascular relaxation in response to acetylcholine was blunted in DH rats. Neither Capto nor RBX treatment restored the endothelium-dependent vascular relaxation (Figure 4A and Table 2). *Ex vivo *incubation of femoral arteries from control rats with insulin induced dose-response vasorelaxation. Insulin failed to induce vasorelaxation in the DH rat arteries. Capto and RBX treatment were able to restore vasodilatory response to insulin (Figure 4B). These results indicate that IUPD followed by high salt diet lead to endothelial insulin resistance at least partially mediated by Renin Angiotensin System (RAS) and PKC signaling.

**Table 2:** Maximal vasorelaxations and concentrations elicited half-maximal vasorelaxation to acetylcholine (ACh), insulin, ACh with insulin, and sodium nitroprusside (SNP)

	Ach		Insulin		Ach + Insulin		SNP	
	
Groups	Max, % IC_50_(× 10^-7^M)		Max, % IC_50_(× 10^-10^M)		Max, % IC_50_(× 10^-7^M)		Max, % IC_50_(× 10^-8^M)	
SD+V	116 ± 22	1.2 ± 0.8	17 ± 12	1.1 ± 1.2	116 ± 17	0.6 ± 0.5	103 ± 20	2.4 ± 1.7
H+V	67 ± 13*	1.3 ± 0.7	6 ± 7*	140+122*	98 ± 15	0.7 ± 0.6	83 ± 17	43 ± 3.1
H+C	65 ± 15*	1.8 ± 0.9	21 ± 10†	3.3 ± 3.8†	88 ± 13	1.3 ± 0.9	83 ± 16	1.7 ± 0.9
H+R	73 ± 14*	1.6 ± 0.9	22 ± 11†	1.5 ± 1.8†	98 ± 11	1.4 ± 0.9	88 ± 15	2.2 ± 1.2

Notes: SD+V: Vehicle treated Sprague-Dawley rats. H+V: Vehicle treated DH rats. H+C: Captopril treated DH rats. H+R: ruboxistaurin treated DH rats. * indicates statistical difference in comparison with SD+V (P < 0.05). † indicates statistical difference in comparison with H+V (P < 0.05).

**Figure 5:**
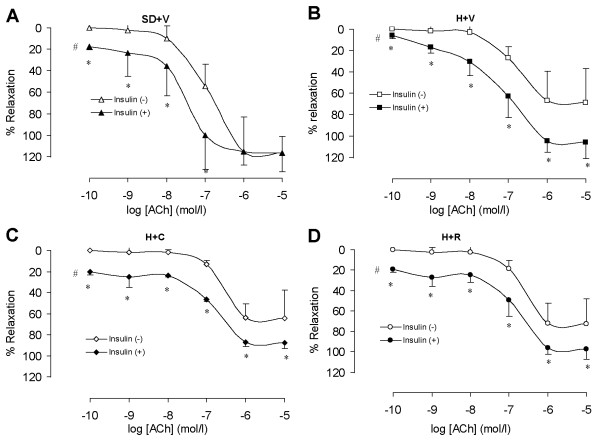
**Endothelium-dependent vasorelaxation with presence of insulin**. A: Vehicle treated SD rats. The presence of insulin increased the vasorelaxation at low doses (P < 0.05). SD+V: Vehicle treated Sprague-Dawley rats. H+V: Vehicle treated DH rats. H+C: Capto treated DH rats. B: Vehicle treated DH rats. The presence of insulin increased the vasorelaxation at most doses (P < 0.05). C: Capto treated DH rats. The presence of insulin increased the vasorelaxation at every dose (P < 0.05). D: RBX treated DH rats. The presence of insulin increased the vasorelaxation at every dose (P < 0.05). n = 7; *: P < 0.05 vs. without presence of insulin at the doses. #: P < 0.05 vs. without presence of insulin using ANOVA.

Surprisingly, in the presence of insulin, endothelium-dependent vascular relaxation in response to ACh was fully restored in all experimental groups (Figure 5 and Table 2). To the best of our knowledge, this is a new phenomenon that is not completely understood and requires further study.

### SMC function

**Figure 6:**
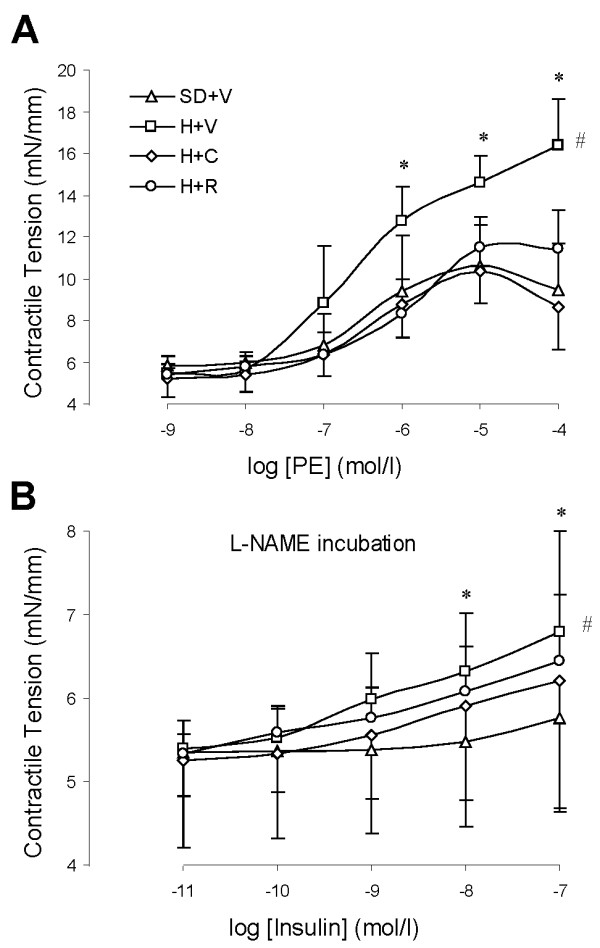
**Vascular contractile tension during vasoconstriction in response to α-adrenergic receptor agonist (phenylephrine, PE) and insulin**. A: PE-induced vascular contraction. SD+V: Vehicle treated Sprague-Dawley rats. H+V: Vehicle treated IUPD hypertensive rats. H+C: Captopril treated IUPD hypertensive rats. B: Insulin-induced vascular contraction while eNOS was blocked with L-NAME. n = 6; *: P < 0.05 vs. vehicle treated of SD rats at the doses. #: P < 0.05 vs. vehicle treated SD rats using ANOVA.

Endothelium-independent vascular relaxation in response to exogenous NO (sodium nitroprusside) was blunted in the DH rats. Treatment with either Capto or RBX did not significantly affect response to exogenous NO (Figure 4C and Table 2). Vascular contraction in response to α-adrenergic agonist (phenylephrine) was enhanced in the DH rats. Both Capto and RBX treatment suppressed vascular contraction in response to phenylephrine (Figure 6A and Table 3). These findings are consistent with SMC functional change concomitant with but independent of endothelial dysfunction.

**Table 3:** Maximal constriction and concentrations elicited half-maximal vasoconstriction to phenylephrine and insulin with L-NAME

	Phenylephrine		Insulin + L-NAME		60 mM KCl
		
Groups	Max, % EC_50_(× 10^-7^M)		Max, % EC_50_(× 10^-6^M)		Max, %
SD+V	9.5 ± 2.2	2.5 ± 0.5	5.8 ± 1.1	3.9 ± 2.2	12 ± 3.1
H+C	16 ± 2.2*	3.1 ± 0.6	6.8 ± 1.2*	0.011 ± 0.035*	13 ± 2.4
H+C	8.7 ± 2.1†	2.3 ± 0.4	6.2 ± 1.6	0.006 ± 0.009*	12 ± 1.2
H+R	11 ± 1.9†	11 ± 3*†	6.4 ± 0.8	6.3 ± 5.2†	13 ± 0.9

Notes: SD+V: Vehicle treated Sprague-Dawley rats. H+V: Vehicle treated DH rats. H+C: Captopril treated DH rats. H+R: ruboxistaurin treated DH rats. * indicates statistical difference in comparison with SD+V (P < 0.05). † indicates statistical difference in comparison with H+V (P < 0.05).

**Figure 7:**
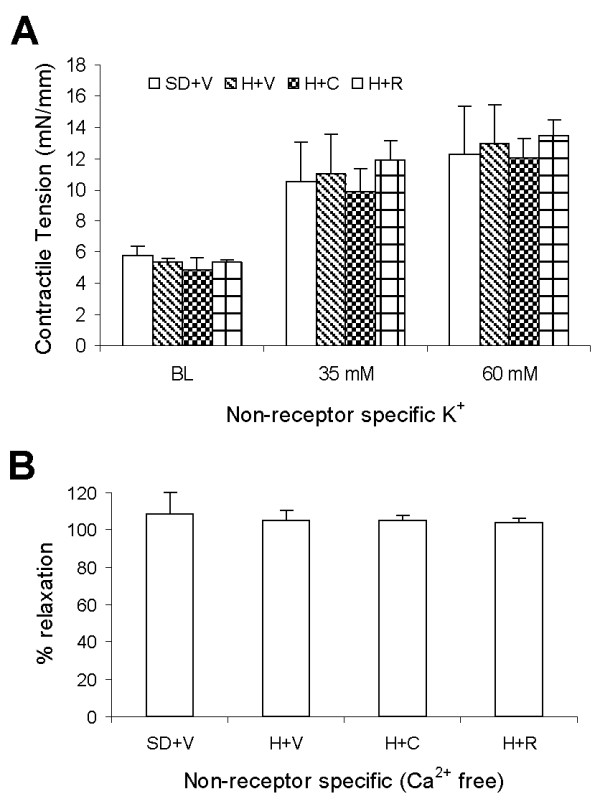
**Non-receptor dependent vasoreactivity**. A: Vascular constriction in response to potassium chloride (KCl). SD+V: Vehicle treated Sprague-Dawley rats. H+V: Vehicle treated DH rats. There was no statistical difference between groups (P > 0.05). H+C: Capto treated IUPD hypertensive rats. B: Vasorelaxation in response to Calcium absent HEPES-PSS while vessel was pre-constricted with phenylephrine (PE). There was no statistical difference between groups (P > 0.05).

We also analyzed direct, NO-independent, effects of insulin on SMC by blocking NO with L-NAME (Figure 6B and Table 3). Interestingly, insulin induced paradoxical vasoconstriction in the presence L-NAME in all groups. When NO was blocked, the vascular contraction was amplified in the DH rats and treatment with Capto and RBX did not significantly suppress insulin-induced vascular contraction (Figure 6B). Finally, we tested the non-receptor dependent vasoreactivity to normalize SMC function. Dose of 35 and 60 mmol/L KCl HEPES-PSS were applied to induce vascular contraction and Calcium (Ca^2+^)-free HEPES-PSS with EGTA (3 mmol/L, Ca^2+ ^chelator) was used to induced vascular relaxation. The KCl-induced vascular contraction was not significantly different in various groups (Figure 7A and Table 3). The Ca^2+ ^free induced vascular relaxation was also not significantly different in various groups (Figure 7B). This confirms that endothelial dysfunction and SMC functional changes found in IUPD rats are not attributed to technical artifacts.

## Discussion

The major finding of this study is that PKC mediates endothelial insulin resistance and vascular smooth muscle hypersensitivity to insulin in DH rats. PKC, however, does not seem to be involved in acetylcholine-mediated vasodilation. The implications and limitations of these findings are elaborated.

Endothelial dysfunction (impaired endothelium-dependent vasorelaxation in response to blood shear stress or acetylcholine) is a salient feature of patients with metabolic syndrome, type 2 diabetes, and hypertension [2,3]. The epidemiologic observations that smaller size at birth is associated with increased rates of coronary heart disease, stroke, type 2 diabetes, adiposity, and metabolic syndrome in adult life have been extensively replicated [15]. IUPD rat is widely used as an animal model of developmentally programmed metabolic syndrome [16]. Endothelial dysfunction has also been described in the IUPD rat model [17]. In the current study, we used the rat model that combined IUPD and postnatal exposure to high salt and have extended observations of endothelial dysfunction to these conditions [10,11,18]. Moreover, we demonstrated that this endothelial dysfunction due to impaired acetylcholine-dependent vasodilation was accompanied by endothelial insulin resistance and SMC hyper contractility in response to both phenylephrine and insulin.

From vascular function point of view, endothelial insulin resistance is manifested by paradoxical contraction of the artery in response to insulin stimulation. Intracellular insulin signaling is mediated by two major pathways, Phosphoinositide (PI)3-kinase/Akt and mitogen-activated protein kinase (MAPK). The former induces NO production and, therefore, leads to vasorelaxation [3]. The latter, however, is associated with the downstream release of endothelin 1 (ET1) that is consistent with vasoconstriction. In the insulin-sensitive endothelial cells, these pathways are well balanced, and insulin stimulation is usually associated with weak vasodilation. Insulin resistance is known to be pathway-selective; i.e., it affects primarily PI3-kinase signaling and either spares or even enhances MAPK/ET1 signaling [3]. As a result, the net effect of insulin is swayed towards vasoconstriction.

It has been demonstrated that endothelial insulin resistance in other animal models of metabolic syndrome, e.g., Zucker fatty rats, diabetic mice, and neonatal rats with high glucose, was mediated by PKC-β [4,18-20]. Most likely, PKC induces insulin resistance by serine phosphorylation of insulin receptor substrate(s) that prevents insulin-induced tyrosine phosphorylation and thus blocks insulin signaling [21]. RBX treatment restored Akt phosphorylation and NO production in response to insulin in the Zucker fatty rats [4]. Our functional data based on pharmacological inhibition of PKC by RBX indicate that PKC activation is also critical for endothelial insulin resistance in the femoral artery of DH rat.

It is unclear how PKC is activated in the arteries of DH rats. Angiotensin II (AngII) type 1 receptor signaling might be the most pertinent in this context [12,22,23]. AngII intracellular signaling involves both calcium and diacylglycerol generation that is consistent with downstream activation of conventional and novel PKC isoforms [22]. AngII is known to induce vascular insulin resistance in a PKC-dependent manner [24]. Our data demonstrated that both Capto and RBX independently conferred vascular benefits. It is unlikely though that angiotensin II signaling is solely mediated by PKC. It is equally unlikely that angiotensin II is the only upstream signal that activates PKC in the vasculature of DH rats. Alternatively, it is plausible that elevated blood glucose and fatty acids are metabolized into diacylglycerol in the vascular wall, and metabolically produced diacylglycerol stimulates PKC [6]. In the current study, we used Capto as a positive control treatment for the blood pressure portion of the study rather than a true mechanistic tool. Future mechanistic studies are necessary to understand possible relationships between AngII-dependent and independent mechanisms of vascular PKC activation.

Surprisingly, we have observed clear dichotomy between drug effects on endothelial insulin resistance and endothelial dysfunction due to impaired acetylcholine-dependent vasodilation. While endothelial insulin resistance was completely ameliorated by Capto and RBX alike, neither compound was able to restore arterial response to acetylcholine. Arguably, this phenomenon may reflect differential effects of complex DH rat milieu on NO production and release. The IUPD rat was diabetic and therefore more complicated than spontaneously hypertensive (SH) rat [25]. It was reported that Capto prevented impaired endothelial response to acetylcholine in SH rats, while antihypertensive treatment did not restore endothelial function in rats with hypertension and diabetes [26-28]. We have also recently shown that restoration of endothelial response to acetylcholine in Zucker diabetic fatty rats required activation of peroxisome proliferation-activated receptor γ, thereby suggesting that this part of endothelial function in diabetic rats is regulated in an angiotensin-independent manner [29]. Future studies are required for deeper mechanistic understanding of this phenomenon.

It is also unclear why exogenous insulin improved endothelial response to acetylcholine in every group. It is possible that insulin may act at the level of NO production. Agonists, including acetylcholine and bradykinin, are known to induce endothelial NO release from eNOS activation mediated in a calcium-dependent manner [30,31]. In diabetes where eNOS activity is impaired, there is less NO to be released in response to a given dose of acetylcholine [1,6]. Insulin stimulates Akt phosphorylation and thereby activates eNOS [1,3]. Therefore, less acetylcholine may be needed to induce vasodilation in the presence of insulin. Phenomenon of insulin-dependent improvement of acetylcholine-induced vasodilation is also reported in the clinic [32,33]. It would be intriguing to study effects of RBX on endothelial insulin sensitivity in the clinic to complement existing data solely focusing on the endothelial dysfunction due to impaired acetylcholine-dependent vasodilation [7-9]. This approach becomes even more appealing in light of new data demonstrating, that in isolated, genetically induced endothelial insulin resistance (mediated by endothelial-specific insulin receptor knock-out), dramatically enhanced development of atherosclerosis in an ApoE-deficient mice model [33].

The current study is the first attempt to address the role of PKC in a very complex non-genomic model related to metabolic syndrome. In follow-up studies, it is necessary to unravel relative contribution of individual risk factors (IUPD, high salt and age) that are known to have direct and indirect effects on vascular function as well as specific PKC isoforms involved in differential vascular response to various stimuli [12,17,34-36]. It is equally important to identify biochemical mechanisms of the observed functional phenomena. Nevertheless, defining PKC as a key regulator of vascular functional response to insulin provides important directions for future experimental and, potentially, clinical studies aiming at preventing vascular complications of metabolic disorders.

## Competing interests

MR and JB are employed by Eli Lilly and ruboxistaurin is a Lilly compound. Other authors have no competing interests.

## Authors' contributions

XL participated in the design of the study, carried out the vascular reactivity experiments, and participated in writing the manuscript. JB participated in the animal studies and physiological data collection. GK participated in the overall design of the study and execution as well as manuscript revisions. MR participated in the design of the study, data collection, and writing the manuscript. All authors read and approved the final manuscript.

## References

[B1] DeFronzo R Insulin resistance, lipotoxicity, type 2 diabetes and atherosclerosis: the missing links. The Claude Bernard Lecture 2009 Diabetologia 2010 53 7 1270 1287 10.1007/s00125-010-1684-1 20361178 PMC2877338

[B2] Tziomalos K Athyros VG Karagiannis A Mikhailidis DP Endothelial dysfunction in metabolic syndrome: Prevalence, pathogenesis and management Nutr Metab Cardiovasc Dis 2010 20 2 140 146 10.1016/j.numecd.2009.08.006 19833491

[B3] Naruse K Rask-Madsen C Takahara N Ha Sw Suzuma K Way KJ Jacobs JRC Clermont AC Ueki K Ohshiro Y Zhang J Goldfine AB King GL Activation of vascular protein kinase C-β inhibits Akt-dependent endothelial nitric oxide synthase function in obesity-associated insulin resistance Diabetes 2006 55 3 691 698 10.2337/diabetes.55.03.06.db05-0771 16505232

[B4] Nuyt AM Mechanisms underlying developmental programming of elevated blood pressure and vascular dysfunction: evidence from human studies and experimental animal models Clinical Science 2008 114 1 1 17 10.1042/CS20070113 18047465

[B5] Schulman IH Zhou MS Jaimes EA Raij L Dissociation between metabolic and vascular insulin resistance in aging Am J Physiol Heart Circ Physiol 2007 293 1 H853 859 10.1152/ajpheart.00138.2007 17434977

[B6] Geraldes P King GL Activation of protein kinase C isoforms and its impact on diabetic complications Circ Res 2010 106 8 1319 1331 10.1161/CIRCRESAHA.110.217117 20431074 PMC2877591

[B7] Beckman JA Goldfine AB Goldin A Prsic A Kim S Creager MA Inhibition of protein kinase C{beta} does not improve endothelial function in type 2 diabetes J Clin Endocrinol Metab 2010 95 8 3783 3787 10.1210/jc.2010-0286 20444914 PMC2913029

[B8] Beckman JA Goldfine AB Gordon MB Garrett LA Creager MA Inhibition of protein kinase C{beta} prevents impaired endothelium-dependent vasodilation caused by hyperglycemia in humans Circ Res 2002 90 1 107 111 10.1161/hh0102.102359 11786526

[B9] Muniyappa R Montagnani M Koh KK Quon MJ Cardiovascular actions of insulin Endocr Rev 2007 28 5 463 491 10.1210/er.2007-0006 17525361

[B10] Mehta NN Sheetz M Price K Comiskey L Amrutia S Iqbal N Mohler ER Reilly MP Selective PKC beta inhibition with ruboxistaurin and endothelial function in type-2 diabetes mellitus Cardiovasc Drugs Ther 2009 23 1 17 24 10.1007/s10557-008-6144-5 18949545 PMC3088108

[B11] Augustyniak RA Singh K Zeldes D Singh M Rossi NF Maternal protein restriction leads to hyperresponsiveness to stress and salt-sensitive hypertension in male offspring Am J Physiol Regul Integr Comp Physiol 2010 298 5 R1375 1382 10.1152/ajpregu.00848.2009 20200128 PMC2867525

[B12] Palkowitz AD Steinberg MI Thrasher KJ Reel JK Hauser KL Zimmerman KM Wiest SA Whitesitt CA Simon RL Structural evolution and pharmacology of a novel series of triacid angiotensin II receptor antagonists Jl Medl Chem 1994 37 26 4508 4521 10.1021/jm00052a010 7799401

[B13] Lu X Kassab G Vasoactivity of Blood vessels using a novel isovolumic myograph Ann Biomed Eng 2007 35 3 356 366 10.1007/s10439-006-9243-0 17221307

[B14] Lu X Kassab GS Assessment of endothelial function of large, medium, and small vessels: A unified myograph Am J Physiol Heart Circ Physiol 2011 300 1 H94 H100 10.1152/ajpheart.00708.2010 21076029 PMC3023250

[B15] Gluckman PD Hanson MA Cooper C Thornburg KL Effect of in utero and early-life conditions on adult health and disease N Engl J Med 2008 359 1 61 73 10.1056/NEJMra0708473 18596274 PMC3923653

[B16] Ogihara T Asano T Ando K Sakoda H Anai M Shojima N Ono H Onishi Y Fujishiro M Abe M Fukushima Y Kikuchi M Fujita T High-salt diet enhances insulin signaling and induces insulin resistance in Dahl salt-sensitive rats Hypertension 2002 40 6 83 89 12105143 10.1161/01.hyp.0000022880.45113.c9

[B17] Brawley L Itoh S Torrens C Barker A Bertram C Poston L Hanson M Dietary protein restriction in pregnancy induces hypertension and vascular defects in rat male offspring Pediatric Research 2003 54 83 90 10.1203/01.PDR.0000065731.00639.02 12646717

[B18] Min W Bin ZW Quan ZB Hui ZJ Sheng FG The signal transduction pathway of PKC/NF-kappa B/c-fos may be involved in the influence of high glucose on the cardiomyocytes of neonatal rats Cardiovasc Diabetol 2009 8 8 10.1186/1475-2840-8-8 19210763 PMC2652442

[B19] Hoyer DP Korkmaz Y Grönke S Addicks K Wettschureck N Offermanns S Reuter H Differential expression of protein kinase C isoforms in coronary arteries of diabetic mice lacking the G-protein Gα11 Cardiovasc Diabetol 2010 9 93 10.1186/1475-2840-9-93 21190563 PMC3024287

[B20] Wei L Yin Z Yuan Y Hwang A Lee A Sun D Li F Di C Zhang R Cao F Wang H A PKC-beta inhibitor treatment reverses cardiac microvascular barrier dysfunction in diabetic rats Microvasc Res 2010 80 158 165 10.1016/j.mvr.2010.01.003 20079359

[B21] Boura-Halfon S Zick Y Phosphorylation of IRS proteins, insulin action, and insulin resistance Am J Physiol Endocrinol Metab 2009 296 4 E581 591 10.1152/ajpendo.90437.2008 18728222

[B22] Wynne BM Chiao CW Webb RC Vascular smooth muscle cell signaling mechanisms for contraction to angiotensin II and endothelin-1 J Am Soc Hypertens 2009 3 2 84 95 10.1016/j.jash.2008.09.002 20161229 PMC2704475

[B23] Pladys P Lahaie I Cambonie G Thibault G Lê NL Abran D Nuyt AM Role of brain and peripheral angiotensin II in hypertension and altered arterial baroreflex programmed during fetal life in rat Pediatr Res 2004 55 6 1042 1049 10.1203/01.PDR.0000127012.37315.36 15071169

[B24] Ketsawatsomkron P Stepp DW Fulton DJ Marrero MB Molecular mechanism of angiotensin II-induced insulin resistance in aortic vascular smooth muscle cells: Roles of protein tyrosine phosphatase-1B Vascul Pharmacol 2010 53 3-4 160 168 10.1016/j.vph.2010.06.001 20601126 PMC4446984

[B25] Manning J Vehaskari VM Postnatal modulation of prenatally programmed hypertension by dietary Na and ACE inhibition Am J Physiol Regul Integr Comp Physiol 2005 288 1 R80 84 15458966 10.1152/ajpregu.00309.2004

[B26] Keaton AK White CR Berecek KH Captopril treatment and its withdrawal prevents impairment of endothelium-dependent responses in the spontaneously hypertensive rat Clin Exp Hypertens 1998 20 8 847 66 10.3109/10641969809053251 9817606

[B27] Lewis SJ Hashmi-Hill MP Owen JR Sandock K Robertson TP Bates JN ACE inhibition restores the vasodilator potency of the endothelium-derived relaxing factor, L-S-nitrosocysteine, in conscious Spontaneously Hypertensive rats Vascul Pharmacol 2006 44 6 491 507 10.1016/j.vph.2006.03.003 16713366

[B28] Ibrahim MA Kanzaki T Yamagata S Satoh N Ueda S Effect of diabetes on aortic nitric oxide synthesis in spontaneously hypertensive rats; does captopril modulate this effect? Life Sci 2005 77 9 1003 14.16 10.1016/j.lfs.2005.02.010 15890370

[B29] Lu X Guo X Karathanasis SK Zimmerman KM Onyia JE Peterson RG Kassab GS Rosiglitazone reverses endothelial dysfunction but not remodeling of femoral artery in Zucker diabetic fatty rats Cardiovasc Diabetol 2010 9 19 10.1186/1475-2840-9-19 20482873 PMC2891691

[B30] Durán WN Breslin JW Sánchez FA The NO cascade, eNOS location, and microvascular permeability Cardiovasc Res 2010 87 2 254 61 10.1093/cvr/cvq139 20462865 PMC2895543

[B31] Fleming I Busse R Signal transduction of eNOS activation Cardiovasc Res 1999 43 3 532 41.11 10.1016/S0008-6363(99)00094-2 10690325

[B32] Rask-Madsen C Ihlemann N Krarup T Christiansen E Kober L Nervil Kistorp C Torp-Pedersen C Insulin therapy improves insulin-stimulated endothelial function in patients with type 2 diabetes and ischemic heart disease Diabetes 2001 50 11 2611 8 10.2337/diabetes.50.11.2611 11679442

[B33] Rask-Madsen C Li Q Freund B Feather D Abramov R Wu IH Chen K Yamamoto-Hiraoka J Goldenbogen J Sotiropoulos KB Clermont A Geraldes P Dall'Osso C Wagers AJ Huang PL Rekhter M Scalia R Kahn CR King GL Loss of insulin signaling in vascular endothelial cells accelerates atherosclerosis in apolipoprotein E null mice Cell Metab 2010 11 5 379 389 10.1016/j.cmet.2010.03.013 20444418 PMC3020149

[B34] Brawley L Poston L Hanson MA Mechanisms underlying the programming of small artery dysfunction: Review of the model using low protein diet in pregnancy in the rat Arch Physiol Biochem 2003 111 1 23 35 12715272 10.1076/apab.111.1.23.15138

[B35] Gu JW Bailey AP Tan W Shparago M Young E Long-term high-salt diet causes hypertension and decreases renal expression of vascular endothelial growth factor in Sprague-Dawley rats J Am Soc Hypertens 2008 2 4 275 285 10.1016/j.jash.2008.03.001 19122855 PMC2598434

[B36] Stewart T Ascani J Craver RD Vehaskari VM Role of postnatal dietary sodium in prenatally programmed hypertension Pediatr Nephrol 2009 24 9 1727 1733 10.1007/s00467-009-1196-8 19421785

